# Crosstalk Between Metabolic Biomarkers and Pulse Wave Analysis in Hypertensive Patients

**DOI:** 10.3390/biomedicines13071514

**Published:** 2025-06-20

**Authors:** Mirela Baba, Mihaela Ioana Maris, Adina Bucur, Daniela Jianu, Simina Mariana Moroz, Dana Stoian, Constantin Tudor Luca, Ioana Mozos

**Affiliations:** 1 Doctoral School, “Victor Babeş” University of Medicine and Pharmacy, 300041 Timisoara, Romania; 2 Center for Translational Research and Systems Medicine, “Victor Babeş” University of Medicine and Pharmacy, 300173 Timisoara, Romania; 3 Department of Functional Sciences-Pathophysiology, “Victor Babeş” University of Medicine and Pharmacy, 300173 Timisoara, Romania; 4 Department of Functional Sciences, Discipline of Public Health, “Victor Babeş” University of Medicine and Pharmacy, Eftimie Murgu Square Nr. 2, 300041 Timisoara, Romania; 5 1st Department of Internal Medicine, “Victor Babeş” University of Medicine and Pharmacy, 300041 Timisoara, Romania; 6 Department of Internal Medicine, Military Hospital, 300080 Timisoara, Romania; 7 Center for Advanced Research in Cardiovascular Pathology and Hemostaeology, “Victor Babeş” University of Medicine and Pharmacy, 300041 Timisoara, Romania; 8 2nd Department of Internal Medicine, “Victor Babeş” University of Medicine and Pharmacy, 300041 Timisoara, Romania; 9 Department of Cardiology, “Victor Babeş” University of Medicine and Pharmacy, 300041 Timisoara, Romania; 10 Department of Cardiology, Institute of Cardiovascular Diseases, 300310 Timisoara, Romania

**Keywords:** arterial stiffness, lipoproteins, metabolic syndrome, insulin resistance

## Abstract

**Background/Objectives:** Hypertension is strongly linked to changes in vascular function and lipid metabolism. This study aimed to examine the relationship between lipid profiles, various metabolic biomarkers, and pulse wave analysis in patients with hypertension. **Methods:** A group of 66 hypertensive patients, aged 64 ± 10 years, participated in pulse wave analysis utilizing an oscillometric device. Multiple lipid serum biomarkers were assessed, such as total cholesterol (TC), triglycerides (TG), and non-HDL cholesterol (non-HDL). Lipid balance index (LBI) was determined by considering TG, LDL, HDL levels, and lipid-lowering medications. **Results:** Notable correlations were observed for SBP, DBP, and early vascular aging (EVA) with lipid biomarkers. In addition to serum lipids, metabolic syndrome, insulin resistance, and non-alcoholic fatty liver disease (NAFLD) were significantly linked to pulse wave analysis variables. Multiple regression analysis showed that only TC continued to have a significant association with DBP. **Conclusions:** Total cholesterol, triglycerides, non-HDL cholesterol, and lipid balance index provide information about systolic and diastolic blood pressure, as well as early vascular aging in hypertensive patients. LBI offers valuable vascular insights in hypertensive individuals with cardiovascular risk factors, early vascular aging, insulin resistance, and NAFLD. The connection between metabolic biomarkers and pulse wave measurements in individuals with hypertension offers a comprehensive method for the early identification of vascular injury and could enhance the prediction of major cardiovascular events.

## 1. Introduction

Hypertension is a major risk factor for cardiovascular diseases, contributing to morbidity and mortality worldwide [1]. It is closely associated with alterations in vascular function and lipid metabolism [2,3,4,5]. Effective management of hypertension requires a comprehensive assessment of vascular health, which extends beyond traditional blood pressure measurements. Pulse wave analysis (PWA) has emerged as a valuable non-invasive tool for evaluating arterial stiffness, providing insights into early vascular dysfunction and predicting cardiovascular events [4,6,7]. It allows the determination of pulse wave velocity (PWV), the velocity of the pulse wave produced during left ventricular ejection throughout the arterial system, augmentation index (AI), including the difference between the reflected and direct wave amplitude, central and peripheral blood pressure variables, and vascular age. As age increases, the elastin levels decrease across the arterial system, and arterial stiffness rises further due to the increased collagen content, proteoglycans, and vascular calcifications [8]. This is the reason why vascular age may be different from the chronological age and can serve as a predictor of future cardiovascular events and all-cause mortality, independently of chronological age [9]. In hypertension, the transmission of pulsatile pressure to microcirculation is enhanced, raising the likelihood of target organ damage [8].

Serum lipid biomarkers play a crucial role in the development of atherosclerosis and endothelial dysfunction [10,11], which are involved in the occurrence of hypertension. Accumulation of LDL cholesterol and other cholesterol-rich lipoproteins in the arterial wall marks the early stages of atherosclerotic plaque formation. HDL cholesterol plays an important role in carrying cholesterol from atherosclerotic plaques back to the liver. Non-HDL cholesterol, calculated as TC − HDL-C, is used in cardiovascular risk assessment: Systemic Coronary Risk Estimation 2 (SCORE2) and SCORE2-Older Persons (SCORE2-OP), in accordance with the latest European Society of Cardiology guidelines on cardiovascular disease prevention in clinical practice [12]. TG analysis is recommended as part of stan-dard lipid analysis.

The interplay between serum lipid biomarkers and PWA parameters has gained increasing attention, as dyslipidemia may contribute to vascular stiffness and impaired hemodynamics in hypertensive individuals [2,3]. One of the main mechanisms is through endothelial dysfunction, in which LDL-C and oxidized LDL impair endothelial nitric oxide (NO) production, which is essential for vasodilation and vascular homeostasis [13,14]. Reduced NO bioavailability leads to increased vascular tone, contributing to increased arterial stiffness and higher pulse wave velocity (PWV), a key parameter in pulse wave analysis (PWA). Another mechanism is via inflammation and oxidative stress [15,16]. Atherogenic lipoproteins stimulate vascular inflammation, increasing levels of cytokines and reactive oxygen species (ROS), enabling degradation of elastin fibers and promoting collagen deposition in the arterial wall, which reduces vascular compliance and increases stiffness. Dyslipidemia can also lead to phenotypic switching of vascular smooth muscle cells (VSMCs) into a more proliferative and synthetic type [17]. This contributes to intimal thickening, fibrosis, and arterial wall stiffening over time.

This study aims to explore the relationship between lipid profiles and pulse wave analysis variables, shedding light on potential mechanisms and clinical implications in hypertensive patients, and to identify metabolic risk factors associated with PWA variables.

## 2. Materials and Methods

### 2.1. Study Population

Hypertensive patients presenting one or more conventional cardiovascular risk factors (hypertension, smoking, diabetes, dyslipidemia, overweight/obesity) were consecutively referred in a primary prevention screening for cardiovascular diseases by family physicians from the western part of Romania to the Institute of Cardiovascular Diseases Timisoara. The patients underwent pulse wave analysis using an oscillometric device. Several lipid serum biomarkers were measured, including total cholesterol (TC), low-density lipoprotein (LDL), high-density lipoprotein (HDL), triglycerides (TG), and non-HDL cholesterol (non-HDL). Lipid ratios such as Atherogenic Coefficient (AC), Atherogenic Index of Plasma (AIP), Castelli Risk Index I (CRI I), Castelli Risk Index II (CRI II), Lipid Index (LI), and Lipid Balance Index (LBI) were also calculated. Information on other laboratory investigations, diagnosis, and therapy was available from the medical records.

Inclusion criteria: Patients presenting one or more conventional cardiovascular risk factors, with hypertension being a mandatory cardiovascular risk factor for inclusion in the study.

Exclusion criteria: Men and women without any conventional cardiovascular risk factors or individuals with a history of established cardiovascular disease (myocardial infarction or stroke).

Ethics Approvals: The investigations were performed considering the principles outlined in the Declaration of Helsinki and have received approval from the Ethics Committee for Scientific Research of the “Victor Babes” University of Medicine and Pharmacy (No. 24/28 March 2022). The informed consent was signed by all study participants, and the aims, procedures, and implications of the study were explained to all of them.

### 2.2. Pulse Wave Analysis

PWV is a measure of arterial stiffness and assesses the speed of travel of the pulse wave generated during left ventricular ejection along the arterial tree. As the pulse wave travels from the aortic root to the peripheral vessels, a first direct systolic wave is produced and, due to changes in diameter and bifurcations, a second reflected wave is produced. The augmentation index (AI) was automatically calculated as the difference between the reflected and direct wave amplitude divided by the pulse pressure and multiplied by 100. Pulse pressure (PP) is the difference between systolic (SBP) and diastolic blood pressure (DBP). Mean arterial pressure (MAP) is calculated as DBP + 1/3(SBP − DBP). The mean of the three readings was used in the current analysis for PWV, AI, and blood pressure. Arterial age is based on PWV in the aorta and was automatically calculated by the Mobil-O-Graph. We considered normal vascular ageing (NVA) if the vascular age corresponded to the chronological age. If the vascular age was lower than the chronological age, the patient was considered to have supernormal vascular ageing (SUPERNOVA). If vascular age was higher than chronological age, the patient had Early Vascular Aging (EVA), associated with an increased risk of cardiovascular events.

The recordings were performed according to the recommendations for standardization of subject conditions [18,19]. Measurements were taken, for each patient, after 10 min of rest, using an appropriate arm cuff, considering arm circumferences, in supine position, with arms extended, in a quiet room, with normal temperature (22 ± 1 °C). Participants were previously asked not to smoke, eat, or drink coffee or alcohol 4 h before the assessment, to keep their eyes closed and not to move or talk during the recording [20].

### 2.3. Serum Lipids

#### 2.3.1. Standard Serum Lipoproteins

The assessment of lipoproteins was performed using the Siemens Dimension clinical chemistry system device. All samples were collected in the morning after a minimum of 8 h of fasting. Total cholesterol (TC), low-density lipoprotein cholesterol (LDL), high-density lipoprotein cholesterol (HDL), non-HDL cholesterol (non-HDL), and triglycerides (TG) levels were assessed. Non-HDL cholesterol was calculated as TC − HDL-C.

#### 2.3.2. Lipid Ratios

Lipid ratios reflect the balance of lipids in the blood and are valuable biomarkers in assessing cardiovascular health [21,22,23,24]. They were calculated, including:

1. AIP—Atherogenic Index of Plasma is calculated as Log TG/HDL-C
2. AC—Atherogenic coefficient or AI—Atherogenic Index is calculated as Non-HDL/HDL
3. CRI I—Castelli Risk Index I is calculated as TC/HDL-C
4. CRI II—Castelli Risk Index II is calculated as LDL-C/HDL-C
5. LI—Lipid Index was calculated, reflecting the pathological or protective effects of serum lipoproteins, adding:
  - (+1) if TC > 200 mg/dL
  - (+1) if LDL > 130 mg/dL
  - (+1) if HDL < 40 mg/dL
  - (−1) if HDL > 50 mg/dL
  - (+1) if TG > 150 mg/dL
  - (+1) if CRI I > 4.5%
6. LBI—Lipid Balance Index was calculated as LI (Lipid Index) − LLD (Lipid Lowering Drugs), LLD = number of lipid-lowering drugs.
  - (+1) Statins,
  - (+1) Fibrates,
  - (+1) Omega 3
  - (+1) Ezetimib

### 2.4. Metabolic Syndrome (MetS)

Metabolic syndrome was defined as the presence of obesity and two of the three following criteria: high blood pressure, impaired glucose metabolism, and elevated non-high-density lipoprotein (non-HDL) cholesterol level (atherogenic dyslipidemia) [25]. Obesity was diagnosed based on abdominal obesity or body mass index (BMI). Abdominal obesity was considered in women with a waist circumference of ≥88 cm and men with a waist circumference of ≥102 cm. Additionally, a body mass index (BMI) higher than 30 kg/m^2^ was used to diagnose obesity. High blood pressure included high normal blood pressure and hypertension, which means SBP > 130 mmHg and DBP > 85 mmHg for office measurement, SBP > 130 mmHg and DBP > 80 mmHg for home measurement, respectively, or anti-hypertensive treatment. Impaired glucose metabolism (prediabetes or diabetes) was considered as glucose ≥ 100 mg/dL or ≥ 140 mg/dL after 120 min in an oral glucose tolerance test or HbA_1_c ≥ 5.7% or on glucose-lowering drug treatment. Atherogenic dyslipidemia was defined as a non-HDL cholesterol level ≥ 130 mg/dL or on lipid-lowering drug treatment.

### 2.5. TyG Index—A Biomarker of Insulin Resistance (IR)

Insulin resistance was assessed using the triglyceride-glucose (TyG) index. The TyG index was calculated using the formula ln [Triglycerides (mg/dL) × Blood glucose (mg/dL)/2]. The cut-off value for insulin resistance was considered >4.5 [26].

### 2.6. Non-Alcoholic Fatty Liver Disease (NAFLD)

Non-alcoholic fatty liver disease (NAFLD) was diagnosed based on the description of mild, moderate, or severe hepatic steatosis in the medical observation records following a previously performed abdominal ultrasound. Evidence of fatty infiltration from ultrasound images included bright hepatic echoes and increased hepatorenal echogenicity. 

### 2.7. Statistical Data Analysis

The statistical processing of the data and sample size calculation was performed using MedCalc^®^ Statistical Software version 23.1.5 (MedCalc Software Ltd., Ostend, Belgium; https://www.medcalc.org; accessed on 4 January 2025). Correlations: Bravais-Pearson correlation index, Spearman’s coefficient of rank correlation, and Kendall rank correlation coefficient (Kendall’s Tau), and also regression analysis were used. A p < 0.05 was considered statistically significant. Normality testing of variables was performed using the Shapiro-Wilk test.

## 3. Results

### 3.1. Characteristics of the Study Participants

The average age of participants was 64 ± 10 years, and 36 patients (55%) were male. The mean BMI was 29.9 ± 5 kg/m^2^, obesity was present in 31 patients (47%), while 55 patients (83%) were either obese or overweight. Smoking was reported in 29 patients (44%). Dyslipidemia was observed in 62 patients (94%), and 19 patients (29%) had diabetes. Metabolic syndrome was present in 28 patients (42%), and the average number of metabolic syndrome (MetS) criteria was 1.43 ± 1.73. Pulse Wave Analysis results, lipid biomarkers levels, biochemical profile, and medication used by patients are presented in Table 1.

### 3.2. Correlations

The Correlations between pulse wave analysis variables and lipid biomarkers and ratios are presented in Table 2.

**Figure 1:**
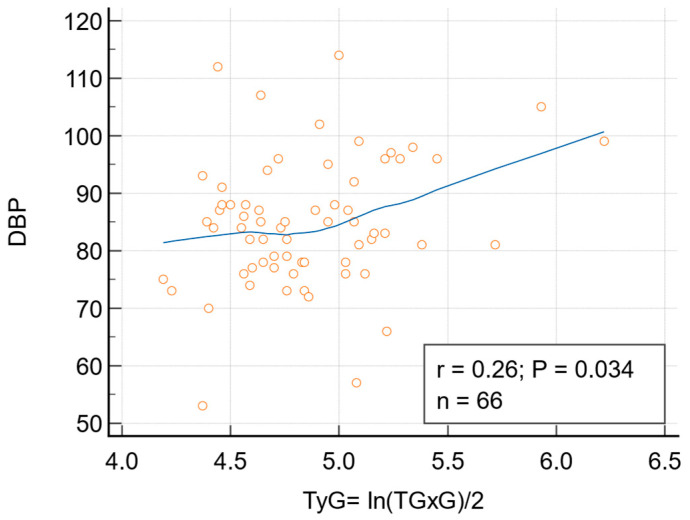
The correlation between diastolic blood pressure (DBP) and triglyceride-glucose index (TyG).

Bravais-Pearson correlations between pulse wave analysis variables and serum lipids and ratios revealed significant correlations only for DBP with TC, non-HDL, and TyG (Table 2). The correlation between DBP and total cholesterol (TC) was significant (r = 0.25, p = 0.042), but the significance was lost after adjusting for lipid-lowering drugs (LLD) (r = 0.2238, p = 0.0731). The correlation between DBP (log) and non-HDL cholesterol (log) was also statistically significant (r = 0.27, p = 0.026, 95% Confidence interval for r: 0.03372 to 0.4836). The correlation remained significant after adjusting for lipid-lowering drugs (LLD) (r = 0.2503, p = 0.044) and BMI and age (r = 0.267, p = 0.032). The correlation between DBP and TyG was statistically significant (r = 0.26, p = 0.034, 95% Confidence interval for r: 0.02100 to 0.4737). The correlation remained significant after adjusting for LLD (r = 0 0.2670, p = 0.0316), but lost its significance after adjusting for age and BMI (Figure 1).

Correlation between DBP and TyG; r = 0.26, p = 0.034, 95% Confidence interval for r: 0.02100 to 0.4737. The correlation remains significant after adjusting for lipid-lowering drugs (LLD): r = 0 0.2670, *p* = 0.0316.

**Figure 2:**
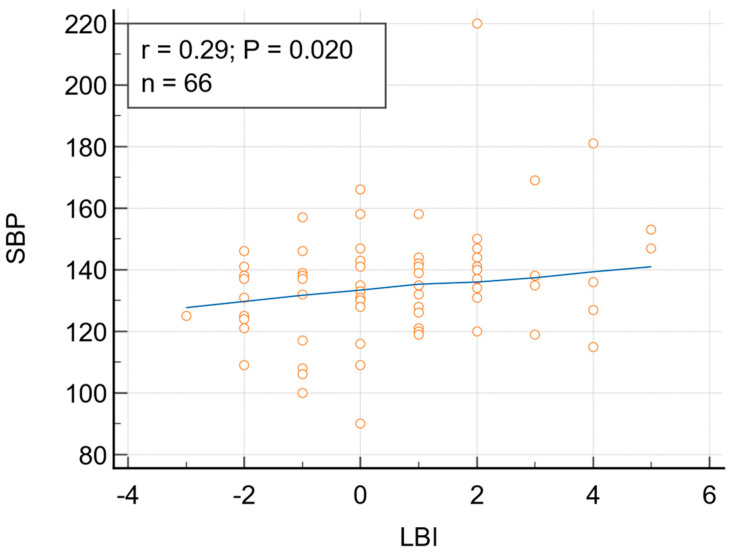
Correlation between Lipid balance index (LBI) and systolic blood pressure (SBP); r = 0.29, p = 0.02, 95% Confidence interval for r: 0.04803 to 0.4945.

Statistically significant correlations were obtained between pulse wave analysis variables and LBI (Table 3). LBI was significantly correlated with SBP (Figure 2) and DBP. The correlation SBP-LBI remained significant after adjusting for age and BMI (r = 0.274, p = 0.0285), but the correlation DBP-LBI lost its significance after adjusting for the same confounders (r = 0.22, p = 0.08). Rank correlation revealed also a significant relationship between SBP and LBI, as follows: Spearman’s coefficient of rank correlation (rho) = 0.263, p = 0.0331, 95% Confidence Interval for rho: 0.0221 to 0.475 and Kendall’s Tau rK = 0.188, p = 0.0259, 95% Confidence Interval for Tau = 0.0252 to 0.337 (Table 4).

The correlation between Lipid balance index and EVA was also very close to significance (r = 0.24, p = 0.053, 95% Confidence interval for r: −0.002516 to 0.4553). Rank correlations between LBI and EVA were also significant (rho = 0.238, p = 0.0548, 95% Confidence Interval for rho: −0.00476 to 0.454 and rK = 0.208, p = 0.014, 95% Confidence Interval for Tau: 0.0192 to 0.389).

Another statistically significant correlation resulted between Lipid index and NAFLD (r = 0.34, p = 0.005, 95% Confidence interval for r: 0.1098 to 0.5400). The correlation remained significant after adjusting for lipid-lowering drugs (LLD) (r = 0.3568, p = 0.0035). The rank correlations were also significant (rho = 0.333, p = 0.0063, 95% Confidence Interval for rho: 0.0990 to 0.532 and rK = 0.294, p = 0.0005, 95% Confidence Interval for Tau: 0.104 to 0.464). The correlation between Lipid balance index and NAFLD was statistically significant (r = 0.29, p = 0.019, 95% Confidence interval for r: 0.04896 to 0.4952). The rank correlation also revealed a significant relationship between NAFLD and LBI, as follows: rho = 0.281, p = 0.0222, 95% Confidence Interval for rho: 0.0421 to 0.490, and rK = 0.246, p = 0.0036, 95% Confidence Interval for Tau: 0.0382 to 0.429.

The correlation between Lipid index and IR was also significant (r = 0.29, p = 0.019, 95% Confidence interval for r: 0.05032 to 0.4962) and remained significant after adjusting for LLD (r = 0.3034, p = 0.0140). Rank correlations were also significant (rho = 0.285, p = 0.0203, 95% Confidence Interval for rho: 0.0464 to 0.493 and rK = 0.252, p = 0.0029, 95% Confidence Interval for Tau: 0.106 to 0.419).

Lipid balance index (LBI) and IR were also significantly correlated (r = 0.25, p = 0.041, 95% Confidence interval for r: 0.01065 to 0.4657). Rank correlations between LBI and IR were also significant (rho = 0.26, p = 0.037 and rK = 0.224, p = 0.0081).

Rank correlations revealed significant correlations of serum lipids, such as TC and TG, with pulse wave analysis variables MAP and EVA (Table 4). PWV was significantly correlated with the number of metabolic syndrome criteria, IR, and NAFLD (Table 4).

### 3.3. Multiple Linear Regression Analysis

A multivariate linear regression analysis (stepwise method) was performed to identify independent predictors for pulse wave analysis variables, IR, NAFLD, and metabolic syndrome. The dependent variables were pulse wave analysis variables, IR, NAFLD, and metabolic syndrome. The results of the multiple linear regression analysis are shown in Table 5.

No variables were retained in the model for EVA, MAP, and PP. TC was found to be a significant determinant of DBP, CRI I > 4.5% was significantly associated with SBP, TyG, and TG, as well as LI was a significant determinant of NAFLD, LI of IR, and PWV of the metabolic syndrome (Table 5). LDL > 130 mg/dL and LBI can be considered determinants of DBP, but the confidence interval is too large. The same observation for the association IR-NAFLD and SBI-LBI (Table 5). The associations in Table 5 remained statistically significant after adjusting for LLD. The relationship between DBP and TC and SBP and CRI I > 4.5%, respectively, remained significant after adjusting for age. Age was found to be a significant determinant of metabolic syndrome after adjusting for SBP, DBP, and PWV (Table 5).

## 4. Discussion

The present study emphasized several significant correlations and associations between pulse wave variables and metabolic biomarkers and uses, for the first time, two new indices: the lipid index and lipid balance index, which consider the pathological values for TC, LDL, HDL, CRI I, and TG and lipid lowering drugs, respectively.

### 4.1. Serum Lipids and Pulse Wave Analysis

In our study, total cholesterol (TC) was independently associated with diastolic blood pressure (DBP). The effect seems to be related to elevated LDL cholesterol levels (>130 mg/dL), according to multiple regression analysis, despite the confidence interval. TC was also associated with mean arterial pressure (MAP) in rank correlations. The Castelli I risk index > 4.5, calculated as TC/HDL-CT, was found to be a significant determinant of SBP. There were no associations of TC with other pulse wave analysis parameters. Several studies have investigated the relationship between total cholesterol levels and blood pressure, including mean arterial pressure, revealing conflicting results. A study involving 107 subjects found a weak but significant positive correlation between total cholesterol levels and systolic blood pressure, and a limited effect on diastolic and mean arterial pressure [27]. Another study examining the correlation between TC and MAP among Africans reported no significant association [28]. However, another study indicated that both systolic and diastolic blood pressure levels increase across varying total cholesterol levels [29]. This suggests a correlation between higher cholesterol levels and increased blood pressure severity. Another recent study involving 63,091 individuals investigated the link between lipid levels and hypertension severity. The findings indicated that elevated TC/HDL-C ratios were associated with higher grades of hypertension, suggesting a potential role of this lipid ratio in blood pressure regulation [30]. However, a study that explored the relationship between total cholesterol, arterial stiffness, and systolic blood pressure suggested that elevated total cholesterol levels are associated with increased arterial stiffness, which in turn contributes to higher systolic blood pressure. The study highlighted the interplay between cholesterol levels and blood pressure regulation [31]. The interplay between blood pressure and cholesterol is mediated by oxidative stress (contributing to endothelial dysfunction and hypertension), cytokines and neuropeptides like angiotensin II, through mechanisms involving bone marrow and microglia activation and genetic factors (such as the endothelial nitric oxide synthase gene variant), all contributing to cardiovascular disease risk [32,33,34,35]. It is worth mentioning that lipids play a significant role in the regulation of arterial blood pressure through several mechanisms involving free fatty acids (can lead to endothelial dysfunction, vascular hypertrophy, and vessel wall stiffness), atrial natriuretic peptide (promotes lipolysis and increases free fatty acid availability), short-chain fatty acids from gut microbial fermentation (interact with specific receptors in blood vessels), and polyunsaturated fatty acids (reducing oxidative stress and regulating vasodilator release) [36,37,38,39].

We also found a significant correlation between non-HDL and DBP. Several studies have investigated the relationship between non-high-density lipoprotein cholesterol (non-HDL-C) and blood pressure, as well as their combined impact on cardiovascular health. In a study focusing on the non-HDL-C to HDL-C ratio (AC—Atherogenic coefficient) as a predictor of hypertension, researchers found a positive association with hypertension. Individuals in the highest AC quartile had a 60% increased risk of developing hypertension compared to those in the lowest quartile. This suggests that a higher AC ratio, reflecting an imbalance between atherogenic and protective lipoproteins, is linked to elevated blood pressure, including increased DBP [40]. A recent study published in Aging Clinical and Experimental Research investigated the association between the non-HDL-C to HDL-C ratio and the onset of hypertension and heart diseases. The researchers found that a higher AC ratio was closely associated with an increased risk of developing hypertension and heart disease [41]. The study also noted that hypertension partially mediated the relationship between elevated AC ratio and heart diseases, suggesting that managing the AC ratio could be crucial in preventing hypertension and subsequent cardiovascular conditions. These studies underscore a significant correlation between non-HDL-C levels and blood pressure. Elevated non-HDL-C and an increased AC ratio are associated with higher blood pressure and an increased risk of hypertension.

We also found a significant correlation between Triglycerides (TG) and Early Vascular Aging (EVA) (rK = 0.171, p = 0.044). Several studies have investigated the relationship between triglyceride levels and EVA, focusing on how elevated triglycerides may contribute to increased arterial stiffness and the early onset of vascular changes. Triglycerides are among the blood lipids most strongly associated with arterial stiffness, and elevated triglyceride levels have been linked to the early stages of cardiovascular diseases, particularly in patients with low LDL-C levels [42]. Elevated triglycerides are a component of atherogenic dyslipidemia, commonly observed in several chronic cardiometabolic disorders. This lipid profile is considered a significant contributor to lipid-dependent residual risk, regardless of LDL-C concentration, and is associated with increased arterial stiffness. It has also been reported that adults with congenital heart disease often experience hypertension and elevated triglyceride levels, identified as positive determinants of early vascular aging in this population [43]. The crosstalk between triglycerides and vascular aging is mediated through genetic factors, oxidative stress, and advanced glycation end products, and is associated with markers of small cerebral vessel disease, predictors of stroke, and dementia [44,45,46,47]. These findings suggest that elevated triglyceride levels are correlated with early vascular aging and increased arterial stiffness. Monitoring and managing triglyceride levels may be crucial in preventing or mitigating the progression of vascular aging and associated cardiovascular risks.

Lipid ratios can be important markers in arterial stiffness because they reflect the balance of lipids in the blood, which can influence the health of the arteries. In general, higher ratios of atherogenic lipids (LDL, triglycerides, etc.) to protective lipids (HDL) are associated with worse arterial health, including increased arterial stiffness [48]. Lipid ratios can also be useful tools in assessing cardiovascular risk [49]. No significant correlation was found with lipid ratios, such as AIP, AC, and CRI I.

Lipid Index (LI) and Lipid Balance Index (LBI) are new indices proposed by the present paper, considering pathological values of TC, LDL, HDL, TG, CRI I, and lipid-lowering drugs, respectively. LBI showed a significant correlation with both systolic (r = 0.29, p = 0.02) and diastolic blood pressure (r = 0.24, p = 0.005), respectively. Moreover, LBI was significantly correlated with EVA (early vascular aging) (rK = 0.201, p = 0.018) and with IR (Insulin Resistance) (r = 0.25, p = 0.041). LI was also correlated with IR (rho = 0.285, p = 0.0203); rK = 0.252, p = 0.0029). Multiple regression analysis revealed significant associations between LBI and peripheral blood pressure values. These results suggest that the new indices are promising in finding new markers of pulse wave analysis, early vascular aging, and insulin resistance. It is useful to include all pathological serum lipids and lipid-lowering drugs in calculating an index related to dyslipidemia.

### 4.2. TyG Index—A Biomarker of Insulin Resistance (IR) and Pulse Wave Analysis

Insulin resistance (IR) is a critical metabolic condition that plays a central role in the development of various chronic diseases, particularly type 2 diabetes, cardiovascular disease, and metabolic syndrome. TyG is a low-cost insulin resistance parameter and widely studied in metabolic syndrome disorders and cardiovascular disease [50]. TyG is also an effective biomarker in identifying non-alcoholic fatty liver disease (NAFLD) [51].

The present study found a significant correlation between TyG and SBP (rK = 0.169, p = 0.0457) and DBP (r = 0.2617, p = 0.0338). The presence of insulin resistance (IR) was correlated with the pulse wave velocity (PWV) (rk = −0.179, p = 0.033) and TyG Index with EVA (Early Vascular Aging) (rK = 0.193, p = 0.0224). Lipid index was a significant determinant of IR, according to multiple regression analysis.

Several studies have explored the relationship between the triglyceride-glucose (TyG) index and blood pressure parameters, demonstrating a positive correlation between higher TyG index values and elevated SBP and DBP levels. A longitudinal study involving 57,192 participants found that each one-unit increase in the TyG index was associated with a 1.93 mmHg rise in SBP and a 1.78 mmHg rise in DBP [52]. This indicates a direct, dose-dependent relationship between the TyG index and both SBP and DBP. Individuals with higher TyG index values had a higher prevalence of elevated blood pressure [53]. Specifically, participants in the highest TyG index quartile exhibited significantly higher SBP and DBP compared to those in the lowest quartile. Several studies have investigated the relationship between insulin resistance (IR) and arterial stiffness, commonly measured by pulse wave velocity (PWV), consistently demonstrating a positive correlation between increased IR and elevated PWV. A study analyzing data from 8,513 adults with hypertension assessed the association between the TyG index and arterial stiffness, as measured by estimated PWV (ePWV), indicating that higher TyG index values were significantly associated with increased ePWV, and highlighting a link between insulin resistance and arterial stiffness in individuals with high blood pressure [54]. A recent study demonstrated that arterial stiffness, measured by brachial-ankle PWV (baPWV), partially mediated the association between insulin resistance and incident atherosclerotic cardiovascular disease (ASCVD). Specifically, 11.1% of the relationship between the TyG index and ASCVD was explained by increased baPWV, underscoring the role of arterial stiffness in the link between insulin resistance and cardiovascular risk [55]. Healthcare professionals may consider incorporating the TyG, LI, and LBI into routine assessments to identify individuals at risk of atherosclerotic cardiovascular disease.

### 4.3. Metabolic Syndrome and Arterial Stiffness

Metabolic Syndrome is a cluster of conditions that increase the risk of cardiovascular disease. In our study, 83% of the patients were obese or overweight, and all the patients were hypertensive; 36% had diabetes or prediabetes, 94% had dyslipidemia, with a total of 42% having metabolic syndrome. We found a significant correlation between metabolic syndrome and PWV in rank correlations (rho = −0.33, p = 0.007), and the correlation remained significant in the multiple linear regression analysis.

Several studies have explored the relationship between metabolic syndrome (MetS) and pulse wave velocity (PWV). A study including 5,181 Chinese participants, aged 40 and above, showed that individuals with MetS had higher brachial-ankle PWV (baPWV) values compared to those without MetS [56]. Moreover, the baPWV increased progressively with the number of present MetS components, highlighting a dose-response relationship between MetS severity and arterial stiffness. Another prospective study evaluated estimated PWV (ePWV) as a potential predictor for the development of MetS in middle-aged adults [57]. Over a median follow-up of 15.6 years, 44.1% of participants developed MetS. Higher baseline ePWV values were associated with an increased risk of developing MetS, suggesting that arterial stiffness may precede and potentially contribute to the onset of MetS. A study using magnetic resonance imaging (MRI) assessed aortic PWV and left ventricular (LV) diastolic function in subjects with and without MetS [58]. Results indicated that those with MetS had increased aortic PWV and decreased LV diastolic function. Interestingly, HDL cholesterol levels were independently associated with both aortic PWV and LV diastolic function, suggesting a link between lipid metabolism and arterial stiffness in MetS patients. A cohort study involving 5,829 middle-aged individuals with MetS but no overt cardiovascular disease found that increased aortic PWV (aPWV) significantly predicted cardiovascular mortality [59]. This suggests that aPWV is a valuable marker for assessing cardiovascular risk in MetS patients. A review highlights that the management of hypertension must be tailored depending on whether it coexists with diabetes mellitus and/or MetS [60]. These conditions significantly influence arterial stiffness and other aspects of pulsatile hemodynamics. In individuals with both hypertension and diabetes, while reducing SBP remains essential, it becomes equally important to avoid excessively lowering DBP. This is because low DBP—especially in elderly patients—can independently increase the risk of cardiac complications.

MetS contributes to increased arterial stiffness through various mechanisms such as endothelial dysfunction, hypertension-induced arterial remodeling, chronic inflammation, and oxidative stress [56,61]. These pathophysiological changes collectively lead to elevated PWV, underscoring the importance of managing MetS components to maintain vascular health. Elevated blood pressure is probably the most significant contributor to increased baPWV, highlighting the role of hypertension-induced arterial remodeling in enhancing arterial stiffness [56]. MetS is associated with increased arterial stiffness, also due to chronic low-grade inflammation and oxidative stress [61]. These factors lead to endothelial dysfunction and structural changes in the arterial wall, such as increased collagen deposition and reduced elastin content, thereby elevating PWV. The rapidly evolving field of metabolomics offers a powerful approach to identify a broad range of metabolic intermediates and end products in body fluids that may contribute to the pathogenesis of arterial stiffness (AS) [62]. Over the past decade, research has increasingly supported this concept by demonstrating associations between AS and various circulating metabolites, including acylcarnitines, glycerophospholipids, sphingolipids, and amino acids. A key objective of metabolomics in this context is to elucidate the underlying metabolic pathways driving AS. These pathways may ultimately serve as promising therapeutic targets in the prevention and management of vascular ageing. Another study that investigated the relationship between fatty acid (FA) composition in serum phospholipids, features of MetS, and arterial stiffness revealed that specific components of MetS were significantly associated with variations in serum phospholipid FA profiles [63]. Notably, arterial stiffness showed an additive association with linoleic acid (LA) and dihomo-γ-linolenic acid (DGLA). These results suggest that maintaining sufficient levels of LA—whether through serum concentrations or dietary intake—may offer potential cardiovascular benefits by contributing to the reduction of arterial stiffness and overall cardiovascular risk.

We suggest that PWV should be used as a non-invasive measure to assess vascular damage in MetS patients. Regular monitoring of PWV in MetS patients can help in early cardiovascular risk assessment and management.

### 4.4. The Interplay Between NAFLD, Cholesterol Metabolism, and Insulin Resistance

Since NAFLD is often linked to additional metabolic comorbidities like dyslipidemia, obesity, or type 2 diabetes, it is commonly regarded as the hepatic manifestation of the metabolic syndrome. Apart from its potential to result in liver-related morbidity and mortality, NAFLD is linked to both clinical and subclinical cardiovascular disease (CVD) [64].

In the present study, we found a linear correlation between NAFLD and LI (r = 0.34, p = 0.0048) and LBI (r = 0.29, p = 0.019), respectively. Non-alcoholic fatty liver disease (NAFLD) significantly influences cholesterol metabolism, leading to dyslipidemia characterized by elevated low-density lipoprotein cholesterol (LDL-C), decreased high-density lipoprotein cholesterol (HDL-C), and increased triglycerides. Several studies have explored the mechanisms underlying this association. Cholesterol accumulation in hepatocytes contributes to the progression of NAFLD. Excess cholesterol can lead to lipotoxicity, promoting liver inflammation and fibrosis [65]. Recent studies have identified specific cholesterol metabolism-related genes that may influence NAFLD development. For instance, a comprehensive study utilizing Mendelian randomization combined with transcriptome analysis highlighted novel targets associated with cholesterol metabolism in NAFLD, suggesting that genetic predispositions affecting cholesterol homeostasis can impact NAFLD progression [66]. These insights highlight the complex interplay between cholesterol metabolism and NAFLD, emphasizing the importance of monitoring and managing cholesterol levels to mitigate NAFLD risk and progression.

TyG, TC, and LI were found, in the present study, according to multiple regression analysis, as significant determinants of NAFLD. Non-alcoholic fatty liver disease (NAFLD) was also closely linked to insulin resistance (IR), with IR playing a pivotal role in the disease’s onset and progression. Studies have identified molecular pathways, such as increased hepatic Platelet Derived Growth Factor AA signaling, that mediate liver insulin resistance in obesity-associated type 2 diabetes [67]. The triglyceride-glucose (TyG) index has been extensively studied as a surrogate marker for insulin resistance and its association with non-alcoholic fatty liver disease (NAFLD). The TyG index is not only effective in identifying individuals at risk for NAFLD but also correlates with the severity of hepatic steatosis and the presence of liver fibrosis. A cross-sectional study involving 4,784 Chinese adults demonstrated that higher TyG index values were significantly correlated with increased severity of hepatic steatosis and a higher prevalence of liver fibrosis in NAFLD patients [68]. Studies have found that elevated TyG index levels are linked to an increased risk of cardiovascular diseases in NAFLD patients. For instance, higher TyG index values were associated with a greater incidence of carotid artery stenosis [69] and coronary heart disease among individuals with NAFLD [70]. Several studies have investigated the relationship between triglyceride (TG) levels and non-alcoholic fatty liver disease (NAFLD), highlighting the significant role of TG accumulation in the development and progression of NAFLD. Investigations into the relationship between waist-to-height ratio (WHtR), triglyceride levels, and NAFLD risk have revealed that triglycerides may mediate the association between central adiposity and NAFLD. This finding emphasizes the crosstalk between body fat distribution, lipid metabolism, and liver health [71]. 

### 4.5. Study Limitations and Strengths of the Study

The cross-sectional study design is associated with difficulties in making causal inference and the inability to investigate the temporal relation between outcomes and risk factors. Arterial stiffness and early vascular aging are surrogate endpoints for cardiovascular events, but using them emphasizes the prophylactic character of our study. The sample size is relatively small, but the power analysis demonstrated an appropriate number of patients. Another limitation is related to the selection of study participants, including patients receiving lipid-lowering drugs, which may question the validity of some results. However, the results did not change after adjusting for LLD in the multiple regression analysis, and LLD was included in the LBI. Including a wider range of ages, such as younger individuals, could have strengthened the research findings. Younger individuals were not included because they were rarely referred for this type of assessment. Additionally, the number of older participants was relatively limited, as they more frequently met one of the study’s exclusion criteria, such as a history of myocardial infarction or stroke.

Strengths of the study lie in the recruitment of real-world, middle-aged hypertensive participants, some of them receiving lipid-lowering drugs. Simple, cost-effective, reproducible biomarkers, such as LI and LBI, can provide valuable, noninvasive information related to large vessels, vascular age, and cardiovascular risk stratification in treated hypertensive patients, enabling personalized diagnosis for each patient. Larger follow-up studies are needed in hypertensive patients to confirm the findings of the present study, to demonstrate that LI and LBI are valuable in predicting cardiovascular events, targets within strategies to improve clinical outcomes, and enable the selection of patients requiring more aggressive therapy. Lipid index and Lipid balance index could be valuable in monitoring hypertensive patients with cardiovascular risk factors, early vascular aging, insulin resistance, and NAFLD. Understanding the crosstalk between metabolic biomarkers and pulse wave analysis variables in hypertensive patients can enable the development of targeted therapies to manage hypertension, vascular function, and dyslipidemia effectively.

## 5. Conclusions

Serum lipids, metabolic syndrome, insulin resistance, and NAFLD are significantly associated with variables of pulse wave analysis. Lipid index and Lipid balance index provide valuable vascular information in hypertensive patients with cardiovascular risk factors, early vascular aging, insulin resistance, and NAFLD.

## Figures and Tables

**Table 1:** Characteristics of the patients included in the study (n = 66 patients).

Variable	Intervals
Cardiovascular risk factors	
Age (years)	64 ± 10
Male	36 patients (55%)
BMI (kg/m^2^)	29.9 ± 5
Obesity	31 patients (47%)
Obesity and overweight	55 patients (83%)
Smoking	29 patients (44%)
Dyslipidemia	62 patients (94%)
Diabetes	19 patients (29%)
Diabetes and prediabetes	23 patients (36%)
Metabolic syndrome	28 patients (42%)
Number of MetS criteria	1.43 ± 1.73
NAFLD	48 patients (73%)
Insulin resistance	55 patients (83%)
Pulse wave analysis	
AI (%)	23.49 ± 16.56
PWV (m/s)	9.45 ± 1.48
SBP (mmHg)	135 ± 19
DBP (mmHg)	85 ± 11
PP (mmHg)	51 ± 13
MAP (mmHg)	106 ± 18
EVA	16 patients (24%)
Serum lipids and ratios	
Lipid index	1.32 ± 1.77
Lipid balance index	0.61 ± 1.89
TC (mg/dL)	196.91 ± 51.87
TG (mg/dL)	191.23 ± 213.58
Non HDL (mg/dL)	147.12 ± 51.62
LDL (mg/dL)	122.86 ± 46
HDL (mg/dL)	49.15 ± 13.6
AIP	5.36 ± 11.49
AC	3.29 ± 1.71
CRI I	4.28 ± 1.71
CRI II	2.62 ± 1.16
TyG	4.86 ± 0.39
Biochemical profile	
Glucose (mg/dL)	117 ± 40
HbA_1_c (%)	6.3 ± 1.1
Creatinine (mg/dL)	0.9 ± 0.3
Uric acid (mg/dL)	6.4 ± 1.6
AST (mg/dL)	25 ± 10
ALT (mg/dL)	27 ± 14
Medication	
ACE inhibitors	34 (51.5%)
ARB	18 (27.3%)
Calcium channel antagonists	34 (51.5%)
Loop diuretics	6 (9.1%)
MRA	5 (7.6%)
Thiazide-like diuretics	25 (37.9%)
Beta-blockers	38 (57.6%)
If channel inhibitors	4 (6.1%)
Potassium channel blockers	4 (6.1%)
Centrally active antihypertensives	7 (10.6%)
Nitrates	7 (10.6%)
Metabolic anti-ischemic drugs	5 (7.6%)
Antiplatelet drugs	30 (45.5%)
Anticoagulant drugs	4 (6.1%)
ARNI	2 (3%)
SGLT2 inhibitors	2 (3%)
GLP 1 agonist	1 (1.5%)
Statins	45 (68.2%)
Selective cholesterol- absorbtion inhibitors	5 (7.6%)
Fibrates	7 (10.6%)
Polyunsaturated fats Omega 3	2 (3%)
Biguanides	8 (12.1%)
Sulphonylureas	6 (9%)
Insulin	5 (7.6%)

Abbreviations: BMI = Body mass index, MetS = Metabolic syndrome, NAFLD = non-alcoholic fatty liver disease, AI = Augmentation index, PWV = Pulse wave velocity, SBP = Systolic blood pressure, DBP = Diastolic blood pressure, PP = Pulse pressure, MAP = Mean arterial pressure, EVA = Early vascular ageing, HbA_1_c = Hemoglobin A1c, AST = Aspartate aminotransferase, ALT = Alanine aminotransferase, TC = Total cholesterol, TG = Triglycerides, Non-HDL = Non-high-density lipoproteins, LDL = Low-density lipoproteins, HDL = High-density lipoproteins, AIP = Atherogenic Index of Plasma, AC = Atherogenic coefficient, CRI I = Castelli Risk Index I, CRI II = Castelli Risk Index II, TyG = Triglyceride-glucose index, ACE inhibitors = Angiotensin-converting enzyme inhibitors, ARB = Angiotensin receptor blockers, MRA = Mineralocorticoid receptor antagonists, ARNI = Angiotensin receptor/neprilysin inhibitor, SGLT2 inhibitors = Sodium-glucose co-transporter-2 inhibitors, GLP 1 agonists = Glucagon-like peptide 1 agonists.

**Table 2:** Correlations between pulse wave analysis variables and lipid biomarkers and ratios.

Correlation Between	r(p)
SBP-AC	0.13 (0.29)
DBP-TC	0.25 (0.042)
DBP-non-HDL	0.27 (0.026)
DBP-nonHDL (adjusted for age and BMI)	0.267 (0.032)
DBP-TyG	0.2617 (0.0338)
DBP-TyG (adjusted for age and BMI)	0.207 (0.0995)
MAP-nonHDL	0.21 (0.08)
PP-CRI II	0.08 (0.49)
PP-HDL	−0.10 (0.42)
PWV-CRI I	−0.10 (0.41)
PWV-TG	−0.18 (0.15)
PWV-LDL	0.13 (0.31)
AI-AIP	−0.18 (0.14)

Abbreviations: SBP = Systolic blood pressure, AC = Atherogenic coefficient, r = Bravais-Pearson correlation coefficient, *p* = significance level, DBP—Diastolic blood pressure, TC = Total cholesterol, Non-HDL = Non-high-density lipoproteins, TyG = Triglyceride-glucose index, MAP = Mean arterial pressure, PP = Pulse pressure, CRI II = Castelli Risk Index II, HDL = High-density lipoproteins, PWV = Pulse wave velocity, CRI I = Castelli Risk Index I, TG = Triglycerides, LDL = Low-density lipoproteins, AI = Augmentation index, AIP = Atherogenic Index of Plasma, BMI = Body mass index

**Table 3:** Pearson’s linear correlations between LI and LBI with pulse wave analysis variables, NAFLD, and IR.

Variable	LI (r/p)	LBI (r/p)
AI	−0.13 (0.30)	−0.16 (0.21)
PWV	0.0092 (0.94)	0.04 (0.73)
SBP	0.22 (0.071)	0.29 (0.02)
DBP	0.19 (0.12)	0.24 (0.05)
MAP	0.094 (0.45)	0.16 (0.21)
PP	0.15 (0.214)	0.21 (0.09)
EVA	0.16 (0.19)	0.24 (0.053)
NAFLD	0.34 (0.0048)	0.29 (0.019)
IR	0.29 (0.019)	0.25 (0.041)
Nr. Criteria MetS	0.057 (0.65)	0.008 (0.95)

Abbreviations: AI = Augmentation index, LI = Lipid Index, LBI = Lipid Balance Index, PWV = Pulse wave velocity, SBP = Systolic blood pressure, DBP = Diastolic blood pressure, MAP = Mean arterial pressure, PP = Pulse pressure, EVA = Early vascular ageing, NAFLD = Non-alcoholic fatty liver disease, IR = Insulin Resistance, MetS = Metabolic syndrome.

**Table 4:** Rank correlations.

Correlation Between	r(p)
PWV-MetS	rho = −0.33 (0.007) rK = 0.27 (0.0012)
PWV-IR	rho = −0.216 (0.0821) rk = −0.179 (0.033)
PWV-NAFLD	rho = −0.221 (0.075) rK = −0.183 (0.029)
SBP-LBI	rho = 0.263 (0.0331) rK = 0.188 (0.0259)
SBP-TyG	rho = 0.237 (0.0555) rK = 0.169 (0.0457)
MAP-TC	rho = 0.256 (0.0384) rK = 0.186 (0.0274)
EVA-TG	rho = 0.207 (0.0953) rK = 0.171 (0.044)
EVA-LBI	rho = 0.23 (0.064) rK = 0.201 (0.018)
EVA-TyG	rho = 0.234 (0.059) rK = 0.193 (0.0224)
NAFLD-LI	rho = 0.333 (0.0063) rK = 0.294 (0.0005)
NAFLD-LBI	rho = 0.281 (0.0222) rK = 0.246 (0.0036)
IR-LI	rho = 0.285 (0.0203) rK = 0.252 (0.0029)
IR-LBI	rho = 0.26 (0.037) rK = 0.224 (0.0081)

Abbreviations: PWV = Pulse wave velocity, MetS = Metabolic syndrome, *p* = significance level, rho = Spearman’s rank correlation, rK = Kendall’s tau rank correlation, IR = Insulin resistance, NAFLD = Non-alcoholic fatty liver disease, SBP = Systolic blood pressure, LBI = Lipid Balance Index, TyG = Triglyceride-glucose index, MAP = Mean arterial pressure, TC = Total cholesterol, EVA = Early vascular ageing, TG = Triglycerides, LI = Lipid Index.

**Table 5:** Results of multiple linear regression analysis.

Dependent Variable	Independent Variables	Multiple R	R Square	Adjusted R Square	95% Confidence Interval	Significance
DBP	TC	0.2507	0.06286	0.04822	0.001961 to 0.1076	0.0423
DBP	LDL > 130 mg/dL	0.2816	0.07928	0.06489	0.9594 to 11.9181	0.022
DBP	LBI	0.2424	0.05875	0.04404	0.0006489 to 2.8954	0.0499
SBP	CRI I > 4.5%	0.2915	0.08496	0.07067	2.1562 to 21.7359	0.0176
SBP	LBI	0.2867	0.08222	0.06787	0.4818 to 5.3349	0.0196
IR	LI	0.2888	0.08343	0.06910	0.01054 to 0.1118	0.0187
NAFLD	TyG (*p* < 0.0001) TG (*p* = 0.0001)	0.7433	0.5525	0.7433	1.0028 to 1.6696 −0.00189 to −0.0006916	<0.0001
NAFLD	LI	0.3428	0.1037	0.1037	0.02738 to 0.1461	0.0048
NAFLD	IR	0.7303	0.5333	0.5260	0.6689 to 1.0766	<0.0001
Metabolic syndrome	PWV	0.2780	0.07726	0.2780	−0.1737 to −0.01278	0.0238
Metabolic syndrome	Age	0.3703	0.1371	0.1237	−0.0296 to −0.006813	0.0022

Abbreviations: R square = Coefficient of determination, DBP = Diastolic blood pressure, TC = Total cholesterol, LDL = Low-density lipoproteins, LBI = Lipid Balance Index, SBP = Systolic blood pressure, CRI I = Castelli Risk Index I, IR = Insulin resistance, LI = Lipid Index, NAFLD = Non-alcoholic fatty liver disease, PWV = Pulse wave velocity, TG = Triglycerides, TyG = Triglyceride-glucose index

## Data Availability

The data presented in this study are available on request from the corresponding authors.

## Abbreviations

The following abbreviations are used in this manuscript:

PWA	Pulse wave analysis
PWV	Pulse wave velocity
AI	Augmentation index
LDL	Low-density lipoproteins
HDL	High-density lipoproteins
Non-HDL	Non-high-density lipoproteins
TC	Total cholesterol
SCORE2	Systemic Coronary Risk Estimation 2
SCORE2-OP	Systemic Coronary Risk Estimation 2 Older Persons
TG	Triglycerides
VLDL	Very-low-density lipoproteins
AC	Atherogenic Coefficient
AIP	Atherogenic Index of Plasma
CRI I	Castelli Risk Index I
CRI II	Castelli Risk Index II
LI	Lipid Index
LBI	Lipid Balance Index
PP	Pulse pressure
SBP	Systolic blood pressure
DBP	Diastolic blood pressure
MAP	Mean arterial pressure
NVA	Normal vascular ageing
SUPERNOVA	Supernormal vascular ageing
EVA	Early vascular aging
LLD	Lipid lowering drugs
MetS	Metabolic syndrome
BMI	Body mass index
HbA_1_c	Hemoglobin A_1_c
TyG Index	Triglyceride-glucose Index
IR	Insulin Resistance
NAFLD	Non-alcoholic fatty liver disease
AST	Aspartate aminotransferase
ALT	Alanine aminotransferase
ACE inhibitors	Angiotensin-converting enzyme inhibitors
ARB	Angiotensin receptor blockers
MRA	Mineralocorticoid receptor antagonists
ARNI	Angiotensin receptor/neprilysin inhibitor
SGLT2 inhibitors	Sodium-glucose co-transporter-2 inhibitors
GLP 1 agonists	Glucagon-like peptide 1 agonists
r	Bravais-Pearson correlation coefficient
*p*	Significance level
